# What practices do parents perceive as effective or ineffective in promoting a healthy diet, physical activity, and less sitting in children: parent focus groups

**DOI:** 10.1186/1471-2458-13-1067

**Published:** 2013-11-12

**Authors:** Sara De Lepeleere, Ann DeSmet, Maïté Verloigne, Greet Cardon, Ilse De Bourdeaudhuij

**Affiliations:** 1 Department of Movement and Sport Sciences, Ghent University, Watersportlaan 2, 9000 Ghent, Belgium

**Keywords:** Parenting, Children, Healthy diet, Physical activity, Sedentary behavior, SDT, SCT

## Abstract

**Background:**

To support parents in improving the health of their young children, examples of effective parenting practices for a healthy diet, physical activity (PA) and sedentary behavior (SB) are needed. This study explores perceived effective and ineffective parenting practices in difficult situations concerning raising healthy children and investigates their relationship with Self-Determination Theory (SDT) and Social Cognitive Theory (SCT). The current study is formative work to inform the content of a randomized controlled trial.

**Methods:**

Four focus groups were conducted between June and October 2012 at worksites during lunch break. A total of 21 unrelated parents of primary schoolchildren (6 fathers, 15 mothers) participated. A short written questionnaire introduced typical difficult situations derived from parental anecdotal reports, concerning healthy diet, PA and SB. These situations formed the backbone for the subsequent focus group discussion. In October 2012, discussions were audio-recorded and analyzed in Nvivo to identify key response items using thematic analysis.

**Results:**

Parents experienced explaining why the child should behave healthily, monitoring, being consistent, offering alternatives, reacting empathetically, modeling, motivating, increasing intrinsic value and availability, and using time-out as effective practices, whereas anger was considered ineffective. Opinions were mixed about the effectiveness of giving as much freedom as possible, obliging, rewarding and punishing, and setting rules and agreements. Parenting practices were consistent with principles from both SDT and SCT.

**Conclusions:**

Parents identified numerous perceived effective practices to respond to their child’s health-related behavior. Since many of them coincide with the evidence base and the success of a parenting program depends upon the degree to which parents’ concerns and motivations are integrated into the program design, important opportunities are created for future intervention programs.

## Background

The benefits of a healthy diet, sufficient physical activity (PA) and limited sedentary behavior (SB) in children, have been well documented and include prevention of overweight and obesity, cardiovascular diseases, depression, fear, stress, poor self-image, and improvement of quality of life [1-5]. Despite these benefits, many children do not meet the recommendations for a healthy diet [6,7], sufficient PA [8] or limited SB [9]. Furthermore, children’s dietary patterns [10,11], PA [12,13] and SB [14] track from childhood into adolescence and adulthood. Therefore, consistently intervening in early years across settings, before an obesogenic lifestyle is deeply rooted, is needed [15].

In the literature, an increasing number of studies and reviews highlights the impact of parenting on the development of healthy children [5,16-26]. There is extensive evidence that parents influence their children’s personal behavioral determinants by shaping their attitudes and social norms and by enhancing their children’s self-efficacy in exhibiting a healthy lifestyle. Within this research area, a subdivision is made between the influence of *general* parenting styles and *specific* parenting practices regarding diet, PA and/or SB [22-28]. The commonly used approach in *general* parenting research is based on the work of Maccoby and Martin [27] who described parenting style as a function of two dimensions of parental behavior: the responsiveness of parents to their child’s needs through affectionate and sensitive interactions (involvement), and the attempt to control their child’s behavior through discipline and expectations (strictness). Results from the review of Sleddens et al. [28] suggest that children raised in authoritative homes (high involvement and high strictness) ate more healthily, were more physically active and had lower BMI levels compared to children who were raised with other styles (authoritarian, permissive, neglectful). *Specific* parenting practices include influencing a child’s specific behavior (healthy diet, PA or SB) via e.g. modeling, social support, parental control, availability and rules and agreements [24,29-31]. Recently, many studies were designed to prevent obesity and/or promote health in children through specific dietary, PA and SB changes that involved parents [19,32-39]. Nevertheless, to the best of our knowledge, no studies have been conducted to assess which of these specific practices parents evaluate as effective and achievable to implement. Since the success of a parenting program depends upon the degree to which parents’ concerns and motivations are integrated into the program design [40], the current study aims to address this gap in literature.

Parental practices have also been studied in Self Determination Theory (SDT) and Social Cognitive Theory (SCT) which both support the principles of authoritative parenting. Parenting practices as reacting empathetically, motivating, increasing the intrinsic value of an activity, applying clear rules and agreements, being consistent, modeling, monitoring and offering alternative activities with as much choice as possible, fulfill both the principles of SDT and SCT [41-46]. Besides these shared principles, each theory also emphasizes different aspects of effective parenting practices. According to SDT, all human beings have the fundamental need to feel related, competent, and autonomous in order to develop and function optimally [47]. Relatedness refers to the need to feel connected to others, to be a member of a larger community, to love and care and to be loved and cared for. The feeling of competence covers the belief that one has the means to control his or her own behaviors and is closely related to the concept of self-efficacy. Finally, the need for autonomy represents an individual’s inherent desire to act as the causal agent of one’s own life and act in harmony with one’s inner self [48]. Another important concept in SDT is internalization, the process by which individuals gradually transform certain externally reached beliefs, attitudes or behaviors into personally appreciated ones. As initial uninteresting activities become more internalized, they are performed with a larger feeling of autonomy, psychological freedom or self-determination [49]. SCT on the other hand specifies the unending reciprocal interrelatedness of personal, physical and social environmental, and behavioral factors [50]. Key elements of SCT include outcome expectancies, perceived self-efficacy, social norms, behavioral skills, reinforcement, and environmental factors such as availability [51]. Additionally, SCT focuses on the impact of external contingencies on the individual’s behavior. When receiving a reward or punishment for a certain behavior, a child is more or less likely to repeat that behavior [44].

Since several reviews conclude that a potential barrier to implement an effective parent-focused intervention is a lack of theory-driven research [11,52,53], a second aim of the current study is to investigate the relationship between the perceived effective and ineffective parenting practices and SDT or SCT.

The present study is situated at the developmental stage of the Intervention Mapping Protocol, a problem- and theory-driven protocol that was especially developed to guide the design of evidence-based intervention programs [54]. Since parents report the need for strategies to encourage their children to eat healthy foods and be more physically active [55], the information arising from the current study will be used to inform the content of a randomized controlled trial consisting of a parenting intervention aimed at promoting a healthy diet, PA, and less SB in primary schoolchildren. Focus groups were held with parents to discover perceived effective and ineffective parenting practices related to the three energy-related behaviors and situating them within SDT and/or SCT.

## Methods

### Participants

The study used a convenience sample of parents of primary schoolchildren. Parents were recruited via their workplace to participate in a focus group during lunch break. All workplaces were located in Flanders (i.e. the Dutch speaking part of Belgium) and belonged to two industries (healthcare and marketing). This recruitment method was preferred over e.g. contacting parents through the child’s school, as participation during lunch break on the worksite was considered more convenient for parents than joining a focus group after school time. Doing so, we aimed to minimize selection bias by also reaching those parents who would otherwise not volunteer for a discussion on children’s health. All employees and employers of the workplace who had at least one primary schoolchild were invited to participate, whereby focus groups consisted of parents of different Social Economic Status (SES). SES was measured using the reported educational level of the parent. Low SES was determined as parents having no higher education (highest obtained degree of secondary school or lower education) and medium to high SES as parents having higher education (vocational college, university or post-academic).

### Procedure

The interview guide was pilot tested for relevance and comprehensability in May 2012 and minor textual modifications were made. All data were collected between June and October 2012 by the same two trained researchers. Thirteen workplaces were contacted, of which three agreed to participate (RR = 23%). Reasons to decline were ‘lack of time’ (n=4) and ‘employing too few people in the study’s target group (parents of a primary schoolchild)’ (n=4). Two companies did not reply. The general manager gave permission to invite all employers and employees to participate via information letters. Since recruitment of a sufficient number of participants (six to ten) was difficult, all parents who volunteered, attended the focus groups. In one company, two focus groups (each with six participants) were organized; the remaining two focus groups were held with four and with five participants.

At the start of the focus group sessions, consent forms were filled in, participants’ anonymity and confidentiality were ensured and permission to audiotape was obtained. The discussion occurred in a medium-sized room and lasted approximately one and a half hour. Lunch was provided at each session and participants received a small incentive of the value of 4$ for their participation. Ethical approval was provided by the Ethics Committee of the University Hospital of Ghent.

In order to familiarize parents with the themes, participants completed a brief questionnaire before the onset of the discussion. This questionnaire introduced some challenging parenting situations related to healthy diet, PA and SB, which relied on anecdotal reports (Table 1). In particular, parents were asked 1) if they had already experienced such a situation; 2) if they considered it as a problem; and 3) how they would react if their child behaved that way. Subsequently, a semi-structured questioning route in which the examples of the questionnaire were used as a starting point to stimulate the discussion, was used to guide the group debate (Table 2). Hereby, parents adjusted the hypothetical situations to their own real-lived experiences. The quality of the questioning route was verified in discussion with prominent health researchers. Every theme (healthy diet, PA, and SB) was wound up with the question ‘Are there any further situations you experience as difficult to let your child be enough physically active/to limit your child’s sedentary behavior/to make your child eat healthily?’. After each focus group, the two researchers debriefed, discussed themes, issues and ideas presented in the session, and discoursed differences with earlier focus groups.

**Table 1:** Examples of difficult situations related to healthy diet, PA and SB discussed in the questionnaire

Difficult situations PA	1. Before Thor leaves for the youth movement/sports club, he suddenly says that he does not want to go. For years, he has always enjoyed himself and has been enthusiastic about the activities, but today he protests upon departure.
	2. Frank is about to cycle to school together with his daughter Marie. Marie complains that she is tired and wants to go to school by car.
Difficult situations SB	1. On a Saturday afternoon at 2 p.m., Martine and Peter are doing the housekeeping (cleaning the table, washing the dishes, ironing, hanging out the laundry, …). Their child Stef asks to watch TV.
	2. Luca has been playing for half an hour on his Nintendo. His mother thinks he has played long enough and tells her son to quit, but Luca does not want to stop playing.
Difficult situations healthy diet	1. Wout’s family is having dinner together. Wout finished his potatoes and meat but leaves his vegetables untouched. He refuses to eat the vegetables because he does not like them.
	2. At breakfast, Liza refuses to eat her sandwich because she does not feel hungry.

**Table 2:** Questioning route for the focus groups, used for every given situation considered in the questionnaire

**Main question**	**Sub question**
1. Who has already experienced such a situation with his/her primary schoolchild?	- How do you react in such a situation?
	- Which core terms did you write down in the questionnaire?
2. Which reactions entail that your child does what you asked for (= positive effect)?	- Are you always consistent?
3. According to you, which reactions are ineffective and cause your child to disobey (= negative effect)?	
4. How does your child react to your behavior or your reaction?	
5. What would your child try to get his/her way?	

### Analyses

All focus groups were audiotaped and transcribed to facilitate analysis. Data were coded and analyzed using Thematic Analysis [56] in the qualitative data analysis software Nvivo 10.0. In Thematic Analysis, a coding framework tends to be constructed on the basis of the theoretical interests guiding the research questions, on the basis of salient issues that arise in the text itself, or on the basis of both [56]. Since SDT and SCT are two prominent theories in parenting literature, the focus groups were conducted taking them into account. Furthermore, whereas focus groups are often chosen as a strategy to discover new themes, the aim of the current group discussions was to identify whether parenting strategies, which are already recognized in literature, were perceived as effective or ineffective by parents. Whilst all data were read and considered, the main aim of the analysis was to identify factors that would need to be taken into account when developing a parenting program.

All four focus groups were independently coded by two trained researchers, which gave a full analysis of interrater reliability (ICC=0.87). Differences were discussed until full consensus was reached.

## Results

### Participants

In total, four focus groups were conducted with 21 parents (age range: 34–50 year) of children aged 6 to 12 year. Parents were predominantly mothers (15/21 or 71%) and the number of children per family ranged from one to four, with a mean of two. The majority (15/21 or 71%) of participating parents had a medium-high SES.

### Practices used by parents

Across all focus groups, almost every parent had experienced the challenging situations described in the questionnaire. Firstly, results regarding general parenting practices are reported. Secondly, more specific results concerning the three energy-related behaviors are reported separately. Key quotes for the various parenting practices were included in the corresponding results section; additional examples are shown in Table 3.

**Table 3:** Quotes from focus group discussion

1. General parenting practices	**Anger**
	*“I sometimes really feel sorry for those children! If you hear parents say: ‘You can beat him’. Then you think…” (Father)*
	*“You even sometimes see them beating their children! I already saw some horrible things happening at the school gate…” (Mother)*
2. Physical activity	**Increasing intrinsic value**
	*“Sometimes, walking is boring for a child. Then you try to sing songs or to make walking more fun by playing a game.” (Father)*
	**Obliging**
	*“Our children are member of a sports club and it has already occurred that they say ‘I don’t want to go anymore because he or she doesn’t come’. Then we oblige them and just say ‘We paid for these sport lessons, so you have to go.’” (Mother)*
	**Motivating your child**
	*“If there is an activity of which I know that she really likes to do it and that’s the reason they don’t want to go anymore – that somebody drops out – then we would first try to convince her to persist.” (Father)*
	**Rewarding**
	*“At school, they really stimulate to go to school in an active way. Every year there is a ‘cycling period’. If they come by bike to school in that period and they wear a helmet and a fluorescent vest, they get a stamp… that stamp and the trading stamp book… yeah, I sometimes really have the impression that children need a lot of stimuli and motivation. It all has to be framed and something has to be done with it. A trading stamp book, a lottery, a gift if they win,… I think all those things matter.” (Mother)*
3. Sedentary behavior	**Rules and agreements**
	*“I think that’s comparable to watching TV. Once you forbid it, they want to watch more.” (Mother)*
	**Monitoring**
	*“We use an alarm clock in the kitchen, a kitchen timer, and when it goes off, it’s finished. We really have to do this because otherwise a discussion gets always started. I let my children put the kitchen timer on themselves… And when it goes off and they don’t want to stop, I say: ‘Either you stop right now, or you don’t play on your Nintendo for the rest of the week’. End of discussion.” (Mother)*
	**Offering alternatives**
	*“Or I propose them to help out, and if they did so, then we do something nice together. […] suggesting an alternative, so they don’t have to watch TV. That works for them.” (Mother)*
4. Healthy diet	**Being a role model**
	*“They will prefer a biscuit over a piece of fruit. Actually, as a parent you should set a good example to your child, but yeah, that doesn’t always happens.” (Father)*
	**Obliging**
	*“We just apply the ‘tasting’ rule. They just have to taste. And if they don’t like it, they don’t like it and then they don’t have to finish it. But they just have to taste.” (Mother)*
	**Availability**
	*“… in weekends, and halfway the week, you buy fruit so you always have fruit available. That way they eat fruit.” (Mother)*
	**Using dessert as a reward or punishing by withholding a dessert**
	*“What’s on the plate, has to be finished. And if it’s finished, they get a dessert. If something stays on the plate, no dessert. So, only rarely they don’t finish their plate because they absolutely want a dessert.” (Mother)*

### 1. General parenting practices

#### Giving an explanation

Parents stated that giving the child a reasonable explanation will make the child understand why he/she has to behave that way, which enhances the chance that the child obeys.

“… if you just can explain them what it is all about, they will experience the problem differently.” (Father)

#### Time-out

Several parents voiced that, when their child flies into a temper, they put him/her in isolation for a few minutes until his/her fury has cooled down. Parents also acknowledged the importance of the general applicability of this practice. A time-out does not have to be restricted to the home setting, but can be used in every situation.

“… and if they don’t get their way, you have to put them immediately in the corner. Wherever you are, put them in the corner. They have to stand there for a couple of seconds or minutes. For a young child, it doesn’t have to last that long… We have done it that way and it has borne fruit.” (Father)

#### Anger

Parents expressed worries about punishing your child with physical violence. Getting angry or even violent as a parent was experienced as an ineffective parenting strategy.

### 2. Physical activity

#### Giving as much freedom as possible

‘Giving your child as much freedom as possible appropriate to his/her age’, was a practice mentioned by parents to raise physically active kids. Parents reported examples as letting their child choose the sport he/she wants to practice and permitting their child to cycle to school via the route they agreed upon. Offering simple choices like choosing between walking, cycling or riding a kick-scooter, was another way to make children more enthusiastic about the activity. Participants also stated that as a child gets older, you have to give him/her more freedom and responsibility. As a parent you can assist your child in choosing by giving advice, but eventually, the child has to make the decision.

“They have to decide themselves what the advantages and disadvantages are […] I also think that an eleven year old child has to be taught that she’s responsible for her own decisions and own behavior. As a parent you can just try to offer them the different options.” (Mother)

#### Monitoring and being consistent

Parents also highlighted the importance of monitoring your child. They mentioned that you can give your child as much freedom as possible, but you also have to supervise that your child sticks to the agreed rules. When the child breaks the rules, you should be consistent and a sanction should be given.

“Since this school year, the 5th grade, S. is allowed to go alone by bike to school. I told her that it was ok, but she had to stop […] to go across and always wear a helmet. ‘Yes yes yes’, she was going to do that. The first day, my father […] was watching her from his van. Of course she arrived without wearing her helmet […]. So I said she wasn’t allowed to go to school by bike for four weeks. If she couldn’t keep to the rules, I assumed she wasn’t ready yet to go alone by bike… she cried, cried […] but I brought her to school by car for four weeks and then allowed her to go by bike again.” (Mother)

#### Increasing intrinsic value

Parents stated that making an activity more pleasant can stimulate children to persist. If children are nagging to do PA, parents use practices like accompanying their child in his/her activities and doing things together with their child. Playing outside together, walking together to school, watching the child for a moment in the sports club… were some other given examples to increase the intrinsic value of an activity.

#### Obliging

Some parents mentioned they would oblige their child if he/she is reluctant to go to their sports club or youth movement. They raise arguments like: “my child has chosen this sport him/herself”, “we have paid for these activities”, or “they have to learn to persist”. On the other hand, other parents stated that obliging their child to participate in a certain sport club or youth movement would make him/her unhappy.

#### Empathy

There was a strong consensus that, when the child really refuses to go to the sports club or youth movement, there probably is a more profound problem. In that case, parents stressed the importance of talking to their child about what really is the matter. That way, they get to know the cause of the problem and can search for a solution together with their child.

“If such a situation would occur, I would try to look for ‘Why?’. If they always liked to go and all of a sudden they refuse, there has to be a cause. Something that explains it, like bullying or another external cause why they don’t want to go anymore. Then I would try to listen, ask for an explanation and then eventually stimulate my child to go.” (Father)

#### Motivating your child

When children struggle to go to the sports club or youth movement, parents try to convince them of the positive characteristics of the activity. Furthermore, parents reported that children often have to be stimulated for doing PA.

#### Rewarding

According to some parents, getting a reward at the end of a task/behavior, is a stimulus for a child to persist. Collecting a certain amount of stamps or saving stickers were two examples mentioned of helpful methods to give a child a reward after he/she succeeded.

### 3. Sedentary behavior

#### Rules and agreements

About the use of rules in managing the amount of TV time, inconclusive results were found. Some parents stated they apply strict rules because otherwise their child would watch TV all day. But parents also mentioned that their child is exceptionally allowed to watch more TV on certain special occasions (e.g. if it rains or when they have a day off from school). In contrast, other parents said their child even would want to watch more TV if there were rules, as this would make him/her more focused on it. Therefore, those parents let their child decide him/herself when and for how long he/she watches TV as long as he/she does sufficient other activities. Two parents admitted they sometimes use TV as a means to be able to do the housekeeping. That way, children keep calm and parents can work undisturbed.

“I do adopt very strict rules. It’s very regulated because otherwise they watch TV all day long.” (Mother)

“As long as he listens when I say ‘Stop’, I don’t think it’s necessary to say that he just can watch on certain days or hours.” (Mother)

#### Monitoring

The use of a kitchen timer or alarm clock was suggested as a practice to monitor screen time. When the alarm goes off, the child knows he/she has to stop and a discussion is prevented. By letting the child put on the kitchen timer him/herself, the child gets involved and listens more easily.

#### Offering alternatives

Parents also reported that it is helpful to propose some alternatives when your child asks to watch TV. Parents gave examples such as going outside together, reading a book or comic or playing a party game. Also involving their children in their own activities such as letting them help with cooking, doing the dishes, folding the laundry, … was mentioned as a practice to limit their child’s SB.

### 4. Healthy diet

#### Rules and agreements

Similarly to the use of rules concerning the amount of TV watching, inconclusive results were found for the use of rules in limiting the consumption of soft drinks. Some parents stated they apply strict rules about how much and when their child is allowed to consume soft drinks. But those parents also revealed exceptions to these rules: e.g. when there is a visitor, on a birthday party, when they dine out… Other parents do not apply rules about consuming soft drinks because they are more concerned about their child not drinking sufficiently than about the type of drinks that is consumed. Regarding the consumption of sweets, some parents stated their child even would want to eat more sweets if there were rules about it as this would make him/her more focused on it.

“In our family, the children always drink water, except if we have a visitor. Then they are allowed to consume soft drinks. Or when we dine out, they can choose what they want to drink. And it isn’t water they will choose.” (Mother)

“Once you forbid sweets, they will… ‘I cannot have sweets, I cannot have sweets…’. So if they go somewhere they think immediately ‘I want some sweets!’.” (Mother)

#### Being a role model

Parents were aware that they are important role models for their child’s eating behavior. Not consuming soft drinks near your child, having breakfast together, drinking water at dinner time, eating fruit… were some given examples of being a good role model.

#### Obliging

To ensure that their child eats enough healthy food, some parents oblige hem/her to eat a certain amount of every served nutritional product. When the child refuses to eat it because he/she does not like the food or does not feel hungry, many parents oblige their child just to taste. Other parents stated they would never oblige their child to eat a certain amount of food, since their own consumption also depends on a varying appetite.

#### Availability

Parents also reported the importance of having healthy food at home. When fruit and vegetables are available in their home, parents have the impression that children consume more of them. Therefore, a good planning of doing the groceries is considered essential.

#### Giving as much freedom as possible

Across all focus groups, there were mixed opinions about taking the child’s food preferences into account. Some parents do not serve dishes that their child dislikes, while others believe a child should learn to taste every single food product. Regarding breakfast, the majority of parents lets their child choose what he/she wants to eat because they find it important he/she has breakfast.

“There is bread or sometimes sandwiches or a toast. But mostly they eat cereals. Because I do find it very important that they have breakfast.” (Father)

#### Using dessert as a reward or punishing by withholding a dessert

Many parents use dessert as a reward for their child finishing his/her plate. They report that this rewarding motivates children to eat healthy food because they absolutely want a dessert. Also for good behavior, desserts are given by some parents.

## Discussion

This study examined the use of parenting practices in raising healthy primary schoolchildren in specific situations around healthy diet, PA and SB. The findings provide new insights into which practices parents use and whether they perceive them as effective or ineffective. Furthermore, these perceived effective and ineffective parenting practices are situated within one or both theories (SDT or SCT). To start with, general findings across all three energy-related behaviors will be discussed. Secondly, specific findings concerning the three energy-related behaviors will be considered separately and finally, some limitations of the study will be recognized.

### General parenting practices

Across all focus groups, a strong consensus was found concerning the positive effect of explaining to a child why it is important to engage in healthy behavior. According to SDT, giving the child a reasonable explanation for behaving healthily, will make the child understand why he/she has to act that way. This autonomy-supportive practice enhances the chance that the child will obey.

In the group discussions using time-out appeared to be an effective and commonly used parenting practice. This practice, which is in accordance with the principles of SCT, was also studied by Kaminski et al. [57]. Teaching parents to use time-out was an effective parent training component associated with larger effects on enhancing behavior in children aged 0–7 [57]. According to our knowledge, time-out has not yet been studied as a parenting practice in the scope of SDT. But, bearing in mind the principles of SDT, time-out could yield benefits in particular circumstances for which mainly the way of using time-out is important. If negative emotions gain the upper hand and the child is impervious to reason, then time-out can be predicted as an effective parenting practice when explaining the reason for the use of time-out and restarting the interaction with the child afterwards.

Furthermore, parents reported the counterproductive practice ‘anger’ of which the ineffectiveness is in line with SDT because it frustrates the fundamental basic human need to feel related, competent, and autonomous [42,47].

### Physical activity

Reacting empathetically, motivating your child to be active and increasing the intrinsic value of an activity are all mentioned in the group discussions as effective parenting practices. Their perceived effectiveness is consistent with both the principles of SDT and SCT as these practices are autonomy supportive and encourage pro-social behavior. This effectiveness was also confirmed in several studies on parenting practices for healthy lifestyles. The review of Kaminski et al. [57] about parent training effectiveness demonstrated that increasing positive parent–child interactions and parental emotional communication skills are consistently associated with larger effects on enhancing behavior in children aged 0–7. Moreover, a growing body of research shows that children are more likely to be physically active when their parents encourage and support them to be active [20,58] and participate together in sport or physical activities with them [59,60].

Other parenting practices considered effective, were applying clear rules and agreements, being consistent and parental monitoring. Parents give their children as much freedom as possible (which is autonomy supporting), but simultaneously they impose the necessary limits by making clear agreements, monitoring the behavior of their child and being consistent when the child does not listen. These parenting practices provide structure to a child which fulfills both the principles of SCT and the fundamental need of feeling competent according to SDT [44,61].

Less consensus existed on the effectiveness of obligations. Some parents stated that they give their child the freedom to choose him/herself to go to the sports club or youth movement, whereas others oblige their child to go. In the latter, parents tend to control their child’s behavior (i.e., pressurize them to behave, think or feel in a certain way) rather than support their child’s autonomy as in the first example. Results of a varied number of studies have affirmed that controlling contexts undermine children’s intrinsic motivation [61], which is incompatible with the concepts of SDT to actively support the child’s capacity to be self-initiating and autonomous [62].

Finally, being rewarded at the end of a task or behavior, was reported by parents as a stimulus for the child to persist. Also in the study of Borra et al. [63] parents mentioned that children need instant gratification. If children have to sustain, they need incentives along the way. This practice of rewarding fits with the principles of SCT, of which the fundamental tenet is that moment-to-moment exchanges are crucial: if a child receives an immediate reward for its behavior, then he/she is more likely to repeat this behavior [44]. In the qualitative study of Sebire et al. [64], children believed that rewards for screen viewing reductions would lead to behavior change. However, some reported that removal of rewards was frustrating. This is in accordance with SDT which believes that rewards undermine children’s natural interest in activities and the need for autonomy [65,66]. Rewards induce children to engage in the activity not for their own enjoyment but to earn the rewards. As a result, children feel pressured, disinterested, or disaffected [67]. Furthermore, for activities that children find inherently interesting, receiving expected rewards leads to a decrement in intrinsic motivation. When reward contingencies are absent, spontaneous engagement in the activity decreases [68].

This work nevertheless found that rewards are a popular and frequently used practice by parents. Therefore, the challenge is to teach parents how this practice can be used without controlling behavior with negative results. Introducing praise, helping parents to recognize feelings surrounding praise and the impact upon behavior, minimizing the predictability of rewards and not using promised rewards but only presenting them after the performance of the behavior, are examples used in the Teamplay intervention in which rewards are used in a positive way [26].

### Sedentary behavior

Since the prevention of overweight and obesity by limiting the amount of SB is a relatively new concept in literature and in public health messages, it was expected that most parents would not yet apply restrictions for SB. Indeed, in the current study some parents did not apply TV viewing rules because formal rules were not perceived as necessary (“my child is physically active enough”) or would cause the opposite effect (greater desire to watch TV).

Nevertheless, some parents do apply clear rules about TV time to limit the SB of their children. Rules provide structure to a child which meets the principles of SCT and the need of feeling competent according to SDT [44]. In other studies, setting rules to limit TV time in general or during meals, has been associated with less screen time, less TV viewing or computer use, and less SB in general [69]. Finally, offering children alternative activities with as much choice as possible, can prevent them from watching TV or other screen-time in an autonomy-supporting (SDT) and problem solving way (SCT) [42,43]. But, although some parents reported they involve their children in household chores as an alternative for screen-time, this seems to be challenging for other parents. Especially in times of stress, parents take the view that they can do household chores better and faster if they do them themselves without in involving their child [70]. How parents can truly engage their children in realistic home-based alternatives to screen time will be an interesting challenge for future interventions.

### Healthy diet

Within the use of rules in eating practices, one has to distinguish rules about snacking and soft drinks from rules about the amount of food to be eaten. About the effectiveness of applying these types of rules, inconclusive results were found in the group discussions. Some parents apply strict rules about how much and when to consume soft drinks whilst others do not apply rules because they are more concerned about their child not drinking sufficiently than about the type of drinks that is consumed. The study of Haerens et al. [71], showed that a lack of family rules related to unhealthy food products was associated with higher fat intake in boys. In girls, lack of food rules was further related to lower levels of fruit intake. Additionally, in the study of Pearson et al. [72], family rules were positively associated with children’s fruit and vegetable consumption. Finally, the use of clear rules to limit a child’s consumption of unhealthy food products, provides structure to a child (SCT) and the need of feeling competent (SDT) [44].

Concerning rules about the amount of food that has to be eaten, some parents ensure that their child eats enough healthy food by obliging him/her to eat a certain amount of every served nutritional product. Other parents stated they never oblige their child to eat a certain amount of food, since their own consumption also depends on a varying appetite. According to literature, rules on the amount of food that has to be eaten, teach children to obey and respect rules rather than learning self-control in their eating habits [73]. Studies proved that such coercive or controlling feeding practices in which the parent, rather than the child, makes decisions about how much the child should eat, are ineffective. When parents control their child’s eating, it undermines the child’s natural ability to respond to its own internal hunger and satiety cues, thus establishing maladaptive patterns of eating [44,74]. Consequently, the concepts of SDT to actively support the child’s capacity to be self-initiating and autonomous are not fulfilled [62].

On the other hand, when parents apply a ‘tasting rule’ which means that the child just tries the food but then has the choice whether or not to finish what is on his/her plate, this can also be seen as providing structure to a child (which fits to both SCT and SDT [44,61]). Furthermore, just tasting will not take away a child’s feeling of hunger and therefore does not undermine the child’s natural ability to respond to its satiety feeling and does not subvert the human need to feel autonomous.

The importance of parental food intake and parental modeling on children’s healthy food consumption was both supported in the group discussions and in other qualitative work [72,75,76]. According to SCT [45], individuals learn behaviors by observing others. This observational learning is most prevailing when the person being observed is powerful, respected, or considered to be alike the observer. Furthermore, parental modeling fulfills the fundamental need of feeling related according to SDT [41].

In the focus groups, many parents stated they use dessert as a reward for their child finishing his/her plate or for good behavior. In theory, SCT claims that moment-to-moment exchanges are crucial. Receiving an immediate reward for a behavior, enhances the chance that a child repeats the behavior [44]. However, this concept cannot be applied to parenting practices for healthy eating because many results reported in literature show that rewarding with food is associated with more problematic eating behavior. Using food (usually sweets) as a reward is theorized to make that food more desirable and the food for which the child is rewarded when eating it less desirable [77]. Also, the use of food for non-nutritive purposes, has been linked with a reduced ability to internally regulate one’s own feelings of hunger and satiety [78]. This matches perfectly with the principles of SDT which proposes that rewarding people undermines intrinsic motivation and creates conditions for a net decline in motivation when rewards are subsequently withdrawn.

All the above makes clear that parents do not raise their children in a way that fits only one theory. Parents use a mix of parenting practices, which can be traced back to SDT ánd SCT. The information arising from this study will be used to inform the content of a randomized controlled trial consisting of a parenting intervention aimed at promoting a healthy diet, PA, and less SB in primary schoolchildren. Such an intervention should not push forward or SDT or SCT as the best theory to teach parents appropriate parenting practices. Better is to select the most feasible effective practices for parents from SDT and SCT and combine both theories. Therefore, the results of this study are an important source of information to integrate parents’ concerns, motivations and current habits, since they determine the success of a parenting program [40].

### Limitations

This study was subject to some limitations. Due to recruitment difficulties, focus groups were mostly conducted with less than the conventional group size of six to ten participants. Although the data demonstrated considerable convergence, other important information may be missed. Secondly, because of voluntary participation, only those parents who are most open to talk about the subject may have been recruited. Thirdly, our focus group research used self-reporting practices, which may have led to inconsistency with actual experiences or social desirability bias. Furthermore, responses to hypothetical situations may not fully represent the reality of parent–child communications. A last limitation is that SES was not used as a stratification/recruitment factor. By recruiting participants via their workplace, unemployed parents were not involved in the group discussions. That way, over-representation of parents with medium-high SES is possible. Furthermore, in this study differences between low and medium-high SES parents were not investigated, which is an important issue that can be included in future research. Taken the above mentioned limitations into account, as with most qualitative research, prudence is in order in generalizing our findings to all parents of primary schoolchildren.

## Conclusions

Parents identified numerous perceived effective and ineffective practices to react to their child’s unhealthy behavior. Many of them were consistent with the effectiveness of practices according to literature, whilst others were not. Furthermore, the reported parenting practices fit with either SCT or SDT or both. Therefore, most feasible practices for parents from SDT ánd SCT should be combined instead of pushing or SDT or SCT forward as the best theory in raising healthy children. That way, new parenting programs can increase children’s healthy diet and PA, and reduce their SB to prevent childhood obesity.

## Abbreviations

ICC: Intraclass correlation coefficient; PA: Physical activity; SB: Sedentary behavior; SDT: Self-determination theory; SCT: Social cognitive theory; SES: Social economic status; RR: Response rate.

## Competing interests

Sara De Lepeleere is a recipient of a PhD-scholarship from the Flemish Agency for Care and Health (B/12732/01). Ann DeSmet, Maïté Verloigne, Greet Cardon and Ilse De Bourdeaudhuij have no financial disclosures. The authors declare that they have no competing interests.

## Authors’ contributions

SDL, AD, MV, GC and IDB developed the standardized protocol and the semi-structured questionnaire. SDL and AD conducted the focus group research in the different companies which included guiding the interviews, transcribing the audiotapes and conducting qualitative content analysis on the transcripts. SDL drafted the manuscript. All authors revised the article critically for important intellectual content and approved the final manuscript.

## Pre-publication history

The pre-publication history for this paper can be accessed here:

http://www.biomedcentral.com/1471-2458/13/1067/prepub
